# Psychological theory in an interdisciplinary context: psychological, demographic, health-related, social, and environmental correlates of physical activity in a representative cohort of community-dwelling older adults

**DOI:** 10.1186/1479-5868-10-106

**Published:** 2013-09-08

**Authors:** Falko F Sniehotta, Paul Gellert, Miles D Witham, Peter T Donnan, Iain K Crombie, Marion ET McMurdo

**Affiliations:** 1 Newcastle University, Newcastle, UK; 2 University of Dundee, Dundee, UK

**Keywords:** Physical activity, Older adults, Social ecological model

## Abstract

**Background:**

Physical activity (PA) in older adults is influenced by a range of environmental, demographic, health-related, social, and psychological variables. Social cognitive psychological models assume that all influences on behaviour operate indirectly through the models constructs, i.e., via intention and self-efficacy. We evaluated direct, indirect, and moderating relationships of a broad range of external variables with physical activity levels alongside intention and self-efficacy.

**Methods:**

We performed a cross-sectional survey of a representative and stratified (65–80 and 80+ years; deprived and affluent) sample of 584 community-dwelling people, resident in Scotland. Objectively measured physical activity and questionnaire data were collected.

**Results:**

Self-efficacy showed unique relationships with physical activity, controlling for demographic, mental health, social, environmental, and weather variables separately, but the relationship was not significant when controlling for physical health. Overall, results indicating support for a mediation hypothesis, intention and self-efficacy statistically mediate the relationship of most domain variables with physical activity. Moderation analyses show that the relationship between social cognitions and physical activity was stronger for individuals with better physical health and lower levels of socio-economic deprivation.

**Conclusions:**

Social cognitive variables reflect a range of known environmental, demographic, health-related and social correlates of physical activity, they mediate the relationships of those correlates with physical activity and account for additional variance in physical activity when external correlates are controlled for, except for the physical health domain. The finding that the social cognition-physical activity relationship is higher for participants with better health and higher levels of affluence raises issues for the applicability of social cognitive models to the most disadvantaged older people.

## Background

Psychological theories of human behaviour have been criticised for overemphasising individual and cognitive factors and for failure to appropriately acknowledge role of environmental, social, economic and health-related variables. Here we take an interdisciplinary perspective and investigate the relationship between psychological variables and a range of demographic, health-related, social, and environmental measures taken in the Physical Activity Cohort Scotland (PACS), the largest, oldest and most detailed ageing cohort study in Europe with objective measurement of physical activity.

Social cognitive models and theories hypothesise that behaviour is a function of specific beliefs that individuals hold about the target behaviour. The psychological models most commonly used to predict and explain health behaviours is the theory of planned behaviour [1,2] and the Reasoned Action Approach [3]. These models hypothesise that behaviour is a linear function of one’s intention and one’s belief about own capabilities (self-efficacy or perceived behavioural control in TPB). In accordance with several other, similar social cognitive models, the TPB assumes that all influences on behaviour (e.g., demographic, health-related, social, and environmental; labelled as ‘background factors’) would operate indirectly through the models constructs. This assumption suggests that any influence would affect behaviour by eventually changing intention or self-efficacy and that thus, intention and self-efficacy provide a sufficient explanation for behaviour [2]. A recent systematic review of 237 prospective tests of the TPB proves evidence for its predictive utility [4], explaining 23.9% of variance in physical activity behaviour. Within 44 prospective tests of physical activity self-efficacy (average correlation *r*_c_ = .31, *B* = .42) and intention (*r*_*c*_ = .45, *B* = .11) revealed to have strongest predictive utility among all TPB variables. Regarding objectively measured physical activity, the correlation was slightly lower (self-efficacy *r*_*c*_ = .18; intentions *r*_*c*_ = .30). Despite this evidence, several studies cast doubt on the accuracy of the sufficiency-hypothesis by demonstrating that variables external to the model account for additional variance in intentions or behaviour once the models’ components have been taken into account.

### Deprivation, age, and gender and physical activity

In a study of 4,286 British women aged 60–79 years [5] socioeconomic position and area of residence deprivation were independently associated with physical activity. Adverse socioeconomic position across the life-course is associated with an increased cumulative risk of low physical activity in older women. Using the Scottish index of multiple deprivations, women living in less deprived areas are more likely to be meeting physical activity recommendations compared with those living in the most deprived areas. Among men, there is no clear trend by deprivation, although the lowest percentage meeting the recommended level was in the most deprived quintile. [http://www.scotpho.org.uk/behaviour/physical-activity/data/adults-by-simd-quintile].

In terms of gender differences in physical activity levels in Scotland men are more active compared to women, as 45% of men and 33% of women meeting physical activity guidelines [6]. Looking into the relation of age and physical activity there is a decrease in activity levels as people get older. 22% of the Scottish men between 65–74 years old and 17% of the women in this age group were sufficiently active to meet the recommendations, whereas only 10% of the 75+ men and 7% of the 75+ women met the recommendations [6].

### Deprivation, age, and gender alongside TPB

Rhodes, Blanchard, and Blacklock [7] found in a population sample of *N* = 6,739 adults that mean values of theory of planned behaviour constructs and correlations with physical activity did not differ by age and gender social cognitive correlates of physical activity, with the exception that mean levels of self-efficacy were lower among younger adults than older adults. Moreover, a meta-analytic review of predictive TPB studies in physical activity by Hagger, Chatzisarantis, and Biddle [8] found no significant differences in prediction patterns between younger versus older samples. Overall, the evidence suggests that adapting theoretical models for specific age groups or based on gender may not be necessary, but this needs further investigation.

### Physical and mental health and physical activity

In older age, higher levels of physical activity have consistently been associated with higher levels of physical functioning and – to a less pronounced degree – mental functioning. Evidently the effect of physical activity on health is predominate [9-11]. Other way round, physical health or functional limitations are typically exhibited as perceived difficulty with engaging in physical activities, such as walking [12].

### Physical and mental health alongside TPB

Physical functioning has been found to predict objectively measured physical activity levels among elderly women over and above self-efficacy and environmental factors [13]. Furthermore, there is longitudinal evidence that changes predict changes in self-efficacy, and changes in self-efficacy are associated with changes in physical activity over 6 months [12]. In contrast, for mental health [14] found the relationship between depressive symptoms and physical activity to be fully mediated by the TPB constructs.

### Social and environmental variables and physical activity

A spectrum of dimensions of the social environment have been shown to influence physical activity behaviour; social support and social networks or social isolation (i.e., loneliness), and neighbourhood factors [15] are prominent ones among others. Although the evidence for social support is somewhat inconsistent for a review on the effectiveness of social support interventions, see [16], many studies have found that favourable characteristics of social networks can have positive effects on the adoption and maintenance of physical activity in older adults. That loneliness is an independent risk factor for physical inactivity was shown by Hawkley, Thisted, and Cacioppo [17] in a population-based sample of 229 older adults. Longitudinal analyses over three years revealed that loneliness was associated with diminished odds of physical activity in the next two years, and greater likelihood of transitioning from physical activity to inactivity. Also perceived social neighbourhood factors, such as trustworthy and helpful immediate neighbours [18], but also more environmental neighbourhood factors like local area surroundings, streets in your area, traffic, pedestrian safety, personal safety [19] were seen as related to the level of physical activity. Finally weather related variables might influence physical activity levels. For instance, research by Chan, Ryan, and Tudor-Locke [20] has shown that factors such as seasonal effects, mean temperature, total rainfall, and the amount of snow on the ground impacting objectively measured physical activity. Authors stressed the fact that these effects may be different in people with different levels of motivation or behavioural intentions.

### Social and environmental variables alongside TPB

Emotional and instrumental social support provided by a family member or friend and size of social support network were found to be unique predictors of health goal attainment after controlling for TPB components in adults aged from 27 to 87 years [21]. In contrast, Hamilton and White [22] did not find social support to be independently associated alongside TPB components. Concerning the role of the environment, Rhodes, Brown, and McIntyre [23] found that TPB variables mediated the relationship between environmental features such as retail land-mix use and neighbourhood aesthetics and physical activity.

To sum up, this raises the question of the relative independent contribution of social cognitive measures over and above external variables, or to the question: Are social cognitions independently associated with behaviour over and above theory external variables? It is also possible that individual cognitions make a difference regarding the effects of external variables on behaviour. For example, it is possible that participants with high perceived behavioural control are less susceptible to an environment which is not conducive to physical activity, or, vice versa, that a lack of resources and environmental opportunity renders individual perceptions of control irrelevant.

The aim of this paper is to test three hypotheses:

1. The hypothesis that social cognitions (i.e., intention and self-efficacy) are associated with behaviour independently from demographic, health-related, social, and environmental factors.

2. The hypothesis that intention and self-efficacy fully mediate the association of demographic, health-related, social, and environmental factors on behaviour

3. The hypothesis that intention and self-efficacy moderate the relation of demographic, health-related, social, and environmental factors on physical activity as a relation modifier or moderator.

## Method

### Participants and procedure

We conducted a cross-sectional survey (wave 1 of the prospective Physical Activity Cohort Scotland (PACS)) using a stratified sample of 584 community-dwelling older people aged 65 and over, resident in Tayside, Scotland. Cohort participants were recruited via random sampling from 17 primary care practices; practices were selected to give a range of rural vs. urban and affluent vs. deprived neighbourhoods. Sampling was stratified according to age (65–80 and 80+ years) and deprivation (Scottish Index of Multiple Deprivation score decile 1–4 versus 5–10; http://www.isdscotland.org). Potential participants were excluded from the cohort if they were resident in institutional care, unwilling to participate, wheelchair or bedbound, had cognitive impairment sufficient to prevent written informed consent, or were enrolled in another research study. Potential recruits were allowed to use walking aids. Full details of the cohort recruitment process have been published previously [24].

Those responding positively to the request to take part in the study were telephoned, eligibility was confirmed, and an appointment made for a home visit. We obtained written informed consent from participants and the study was approved by the Tayside Committee on Medical Research Ethics. The study is in compliance with the Declaration of Helsinki (09/S1401/57). We conducted two home visits with each participant, spaced 7 days apart. Details of the questionnaires administered at each visit are given in Table 1. Data were collated, anonymised and processed by the Health Informatics Centre (HIC, http://www.dundee.ac.uk/hic) according to their standard operating procedures. At the baseline visit, we provided participants with an RT3 accelerometer (Stayhealthy inc, Monrovia, California, USA). The RT3 is a piezoelectric, triaxial accelerometer which has previously been shown to discriminate walking from sedentary activity in older people [25], and which is responsive to interventions designed to increase physical activity [26]. Summed activity counts were recorded each minute for 7 days. Daily regional hours of sunlight, rainfall and maximum temperature data were obtained from the Meteorological Office, utilising data collected at the Mylnefield weather station.

**Table 1:** Measures by domain

**Domain**	**Questionnaire**	**Reference**
Demographics	Scottish Index of Multiple Deprivations (SIMD), age and gender	http://www.isdscotland.org
Physical health	SF-36 Health-related quality of life, subscales: Physical functioning, role physical, bodily pain, general health	[27]
	Functional Limitations Profile (FLP): Physical domain	[28]
Mental health	SF-36 Health-related quality of life, subscales: Vitality, social functioning, role emotional, mental health	[27]
	Hospital Anxiety and Depression Score (HADS): Depression, Anxiety	[29]
	Functional Limitations Profile (FLP): psychosocial domain	[28]
Social influences	Need for support and received support	[30]
	Loneliness (R-UCLA loneliness scale)	[31]
	Neighbourliness: Social capital module	[18]
Environmental	Older Peoples Active Living (OPAL) questionnaire: Local area surroundings, streets in your area, traffic, pedestrian safety, personal safety	[19]
Weather	Sun (hours), minimum temperature and maximum temperature (in grad Celsius), rainfall in millimeter	Meteorology records at Mylnefield weather station, sited at the Scottish Crop Research Institute and contributing data via the UK Meteorological Office (http://www.metoffice.gov.uk)
Social cognitions	Extended theory of planned behaviour questionnaire: Intention, self-efficacy	[32]
Physical activity	RT3 accelerometer: Stayhealthy inc, Monrovia, California, USA in mean of daily physical activity recorded over the 7-day period	[25,26]

### Sample size calculations and response rates

We planned a sample size of 600 (150 per stratum) to permit the influence of an individual attribute to be detected with 80% power if the correlation with a particular outcome was as low as 0.11. For multiple regression analyses this sample size would allow consideration of 30 variables with a multiple correlation coefficient of 0.2 or *R*^*2*^ of 4%. It would allow detection of a change in *R*^*2*^ as low as 2.5% in a multiple regression model with the addition of 15 variables to a model with 15 variables already entered with 80% power. Participants were recruited between October 2009 and January 2011 from 17 General Practices.

3343 letters of invitation were sent to potential participants. The deprivation category was unknown for 7/3343 so 3336 was used as the denominator. Overall 63% replied to their invitation (either positively or negatively) and 19% responded positively indicating a willingness to participate. A total of 584 people, age range 65 to 100 years, 46% (268/584) male participated, i.e. 97.3% (584/600) of the target sample size. Accelerometry data were available for 547/584 (93.6%) of participants.

For physical activity, the mean (*SD*) number of minutes of activity per day recorded by accelerometry were highest at 214 (89) for the affluent young old; followed by 192 (84) for the deprived young-old, deprived; then 156 (87) for the affluent old-old; and lowest 128 (68) for the deprived old-old. For the total sample of 547 participants the mean (*SD*) was 177 (89) minutes per day. The mean age was 73 (5) years in both, deprived and affluent young-old and 86 (5) years in the deprived old-old and 85 (4) years in the affluent old-old, resulting in a mean of 79 (8) years in the total sample. Overall, in terms of gender, there were slightly more women (54%) than men in the sample (deprived young-old: 55, deprived old-old: 57; affluent young-old: 54; affluent old-old: 51).

### Measures

Details of the questionnaires administered are given in Table 1, whereas their correlations, means, ranges, and standard deviations were displayed in Table 2.

**Table 2:** **Estimated correlation coefficients, means**, **standard deviations, and range***s*

**Variables**	***Correlation with Intention***	***Correlation with self***-***efficacy***	***Mean***	***SD***	***Range***
Demographic					
Gender (0 = women, 1 = men)	.11**	.12**	54.3% women, 45.7% men		0 – 1
Age	-.18**	-.18**	78.4	7.7	65 – 105
SIMD	.10	.09*	5.2	2.7	1 – 10
Physical health					
SF-36 physical functioning	.51**	.59**	71.7	23.0	5 – 100
SF-36 role physical	.26**	.33**	82.3	25.9	0 – 100
SF-36 bodily pain	.29**	.32**	69.3	26.8	0 – 100
SF-36 general health	.37**	.43**	67.6	21.1	5 – 100
FLP physical domain	-.49**	-.59**	174.6	20.8	0 – 409
Mental health					
SF-36 vitality	.38**	.43**	59.9	20.8	0 – 100
SF-36 social functioning	.24**	.31**	88.2	22.7	0 – 100
SF-36 role emotional	.09*	.12**	96.5	11.6	25 – 100
SF-36 mental health	.22**	.22**	82.8	13.5	25 – 100
FLP psychsocial domain	-.41**	-.49**	188.1	116.1	0 – 462
Depression	-.31**	-.39**	3.7	2.7	0 – 14
Anxiety	-.09*	-.10*	4.2	3.3	0 – 19
Social					
Need for support	-.22**	-.24**	1.7	1.2	1 – 6
Received support	.33**	.36**	2.3	1.6	1 – 6
Loneliness	.16**	.17**	8.2	1.3	3 – 9
Neighbourliness	.13**	.13**	4.7	1.4	0 – 6
Environmental					
Local area Surroundings	20**	.19**	22.3	3.7	10 – 28
Streets in your local area	.11**	.12**	14.2	2.5	6 – 20
Traffic	.08	.09*	11.2	2.7	4 – 16
Pedestrian safety	.08	.10*	19.9	3.3	10 – 28
Personal safety	.31**	.32**	35.7	4.4	19 – 42
Weather					
Sun (hours)	-.04	-.04	29.0	15.5	1.1 – 60.7
Minimum temperature	-.03	-.06	4.4	4.6	-8.9 – 12.2
Maximum temperature	-.03	-.05	12.1	5.3	-2.3 – 22.0
Rainfall	-.07	-.05	18.4	14.3	0 – 74.3

*Note*. *N* = 574. *p < .05, **p < .01. *FLP* Functional limitations profil, *SF-36* Short-Form 36 Health Survey, *SIMD* Scottish Index of Multiple Deprivation.

### Analytical procedure

All hypotheses were tested using regression analytical procedures in SPSS v19. For all coefficient estimations (bivariate correlation, hierarchical regression, mediation, moderation), nonparametric bootstrapping procedure [33] with 5,000 resamples was employed, which allows estimating the sampling distribution of a statistic empirically without making assumptions about the form of the population, and without deriving the sampling distribution explicitly. All variables were z-standardized prior to analyses, which allow direct comparisons of all coefficients between the models tested, working as effect size estimation.

Answering Hypothesis 1, hierarchical regression analyses were used. Each set of external variables was included as predictors in a first step, in a second step the social cognitive variables, intention and self-efficacy respectively, were included additionally.

For investigating Hypothesis 2, a series of simple mediation analyses was conducted (see Table 3) containing three variables per model only: one external variable (predictor), one social cognitive variable (mediator), and physical activity (outcome). In a simple mediation model path coefficient *c* quantifies effect of an external variable on physical activity (total effect), which can be further decomposed into path *a*, representing the effect of the external variable on the proposed mediator (i.e. intention or self-efficacy), path *b* represents the effect of the mediator on physical activity, controlling for the external variable in question, and path c′, which represents the effect of the external variable on the physical activity controlling for the social cognitive mediator (direct effect). We directly tested the significance of indirect effect using a SPSS macro [33] that facilitates estimation of the indirect effect as bootstrapped product of coefficients *ab* in conjunction with bootstrapped 95%-confidence intervals (CI) for inference testing. The null hypothesis of no indirect effect is rejected at the level of significance (*p* > .05) if 0 lies outside the bootstrapped 95%-CI. Since the sampling distribution of *ab* tends to be asymmetric, with nonzero skewness and kurtosis, the primary advantage of bootstrapping is that no assumptions are made about the sampling distributions of *a*, *b*, or their product, because bootstrapping approximates the sampling distribution of *ab* empirically.

**Table 3:** Estimated coefficients for mediation analyses on objectively measured physical activity

**Variables**	**Intention**							**Self**-**efficacy**						
	***a***	***b***	***c***′	***c***	***ab***	***LLCI***	***ULCI***	***a***	***b***	***c***′	***c***	***ab***	***LLCI***	***ULCI***
Demographic														
Gender (0 = women, 1 = men)	.23**	.32***	.08	.16	.08+	.02	.14	.23**	.35***	.07	.17	.08+	.03	.15
Age	-.19***	.25***	-.34***	-.39***	-.05+	-.08	-.03	-.18***	.30***	-.33***	-.39***	-.05+	-.08	-.03
Deprivation	.08	.31***	.14***	.17***	.03	-.001	.05	.09*	.34***	.14***	.17***	.03+	.003	.06
Physical health														
SF-36 physical functioning	.51***	.09*	.45***	.50***	.05+	.01	.09	.59***	.10*	.44***	.49***	.06+	.01	.11
SF-36 role physical	.26***	.28***	.17***	.24***	.07+	.05	.11	.33***	.31***	.14***	.24***	.10+	.07	.14
SF-36 bodily pain	.29***	.30***	.09*	.18***	.09+	.05	.13	.32***	.34***	.07	.18***	.11+	.07	.15
SF-36 general health	.37***	.25***	.21***	.30***	.09+	.06	.13	.43***	.28***	.16***	.30***	.12+	.08	.17
FLP physical domain	-.49***	.14**	-.39***	-.45***	-.06+	-.11	-.03	-.59***	.14**	-.37***	.45***	-.08+	-.14	-.03
Mental health														
SF-36 vitality	.38***	.24***	.20***	.30***	.09+	.06	.13	.43***	.28***	.18***	.30***	.12+	.08	.17
SF-36 social functioning	.24***	.30***	.14***	.21***	.07+	.04	.10	.31***	.33***	.11**	.21***	.10+	.07	.14
SF-36 role emotional	.09*	.32***	.04	.06	.03	-.001	.06	.12**	.35***	.02	.06	.04+	.01	.08
SF-36 mental health	.22***	.31***	.03	.10	.07+	.04	.10	.22***	.35***	.02	.10*	.08+	.05	.12
FLP psychosocial domain	-.41***	.26***	-.16**	-.26***	-.10+	-.15	-.07	-.50***	.30***	-.11**	-.26***	-.15+	-.20	-.11
Depression	-.32***	.25***	-.23***	-.31***	-.08+	-.12	-.05	-.39***	.28***	-.20***	-.31***	-.11+	-.15	-.07
Anxiety	-.09	.33***	.07	.04	.03+	-.06	-.001	-.10*	.37***	.07	.04	.03+	-.07	-.003
Social														
Need for support	-.22***	.31***	-.05	-.12**	-.07+	-.10	-.04	-.25***	.35***	-.03	-.12**	-.09+	-.13	-.07
Received support	.35***	.29***	.09*	.19***	.10+	.07	.14	.34***	.33***	.07	.19***	.12+	.08	.16
Loneliness	.15***	.32***	.09*	.14**	.05+	.02	.08	.17***	.36***	.07	.14	.06+	.03	.10
Neighbourliness	.14**	.31***	.10**	.14***	.04+	.02	.07	.14***	.34***	.10*	.14***	.05+	.02	.08
Environmental														
Local area Surroundings	.20***	.31***	.08**	.14**	.06+	.04	.10	.19***	.34**	.08	.14**	.06+	.04	.10
Streets in your local area	.11*	.32***	-.01	.03	.04+	.01	.07	.12**	.36***	-.01	.03	-.04+	.01	.07
Traffic	.08*	.32***	.01	.03	.03+	.01	.06	.09*	.36***	.001	.03	.03+	.002	.06
Pedestrian safety	.08	.32***	-.03	.01	-.04+	-.002	.05	.01*	.36***	-.04	-.01	-.03+	.01	.07
Personal safety	.31***	.26***	.19***	.27***	.08+	.05	.12	.32***	.30***	.17***	.27***	.10+	.06	.13
Weather														
Sun (hours)	-.04	.32***	.07	.05	-.02	-.04	.01	-.04	.35***	.07	.05	.01	-.05	.01
Minimum temperature	-.03	.32***	.01	.00	-.01	-.04	.02	-.06	.35***	.03	.01	-.02	-.05	.01
Maximum temperature	-.03	.32***	.04	.03	-.01	-.04	.02	-.05	.35***	.04	.03	-.01	-.05	.01
Rainfall	-.07	.31***	-.04	-.06	-.02	-.04	.003	-.05	.35***	.03	.01	-.02	-.05	.01

*Note*. *N* = 574. LLCI and ULCI refer to the lower and upper limit 95%-confidence interval of the indirect effect; + refers to 95%-confidence interval of the point estimated indirect effect does not include zero, i.e. the indirect effect is significant *p* < .05. *FLP* Functional limitations profil, *SF-36* Short-Form 36 Health Survey.

*p < .05, **p < .01, ***p < .001.

Addressing Hypothesis 3 moderation was tested by forming product terms (i.e. one social-cognitive variable with one external variable, z-standardized centred prior to product term forming) that represent the interaction, and estimating the increment in variance explained by the product term after effects of variables the product term contains have been controlled. This, again, was done separately for each moderation hypothesis.

Since bootstrapping resampling cannot be performed for datasets with multiple imputations (MI) in SPSS v19 and since all variables in the final sample (n = 547) had less than 1% missing values (except Loneliness = 5% and Neighbourliness = 2%) no imputation was conducted [34]. Comparing (non-bootstrapped) results for Loneliness scale in imputed (MI; 5 datasets) and in not imputed datasets coefficient estimates were exactly the same, indicating missing data mechanism is ignorable in present case.

For all hierarchical regression analyses for Hypothesis 1 the variance inflation factors ranging between VIF = .44 and .99 and the tolerance statistics between 1/VIF = 1.00 and 2.27, indicate that the variance of the parameter estimates is not inflated to a critical degree [35]. Only for SF-36 physical functioning subscale (1/VIF = .36, VIF = 2.80) the estimated coefficient is slightly elevated.

## Results

Correlations, means, standard deviations, and ranges for all study variables are displayed in Table 1.

### Direct associations of social cognitions and physical activity (hypothesis 1)

Summarising the results for hierarchical regressions, self-efficacy explained additional variance in physical activity, controlling for demographic, mental health, social, environmental, and weather variables separately with the exception of physical health, where no additional variance was accounted for by social cognitions (see Table 4).

**Table 4:** Estimated coefficients for hierarchical regression analyses

	***Step 1***	***R***^***2***^	***Step 2***	**∆*R***^***2***^
Demographic		.19		.07
Gender (0=f, 1=m)	.15*		.09	
Age	-.40***		-.35***	
Deprivation (SIMD)	.18***		.16***	
Intention			.009	
Self-efficacy			.27***	
Physical health		.27		.01
SF-36 physical functioning	.40***		.38***	
SF-36 role physical	-.02		-.009	
SF-36 bodily pain	-.12**		-.12**	
SF-36 general health	.02		.01	
FLP physical domain	-.21***		-.19***	
Intention			.07	
Self-efficacy			.002	
Mental health		.15		.03
SF-36 vitality	.14*		.09	
SF-36 social functioning	.06		.04	
SF-36 role emotional	-.02		-.01	
SF-36 mental health	.03		.01	
FLP psychosocial domain	-.13**		-.05	
Depression	-.20***		.18***	
Anxiety	-.21***		-.17***	
Intention			.05	
Self-efficacy			.18*	
Social		.12		.04
Need for support	-.12**		-.04	
Received support	.18***		.07	
Loneliness	.07		.05	
Neighbourliness	.10*		.09*	
Intention			.04	
Self-efficacy			.28***	
Environmental		21.		.05
Local area Surroundings	.04		.02	
Streets in your local area	.00		-.02	
Traffic	-.07		-.05	
Pedestrian safety	-.06		-.06	
Personal safety	.29***		.20***	
Intention			.02	
Self-efficacy			.29***	
Weather		.01		.13
Sun (hours)	-.01		-.01	
Minimum temperature	-.24		-.17	
Maximum temperature	.26		.19	
Rainfall	-.04		-.02	
Intention			.06	
Self-efficacy			.30***	

*Note*. *N* = 574. *p < .05, **p < .01, ***p < .001. *FLP* Functional limitations profil, *SF-36* Short-Form 36 Health Survey, *SIMD* Scottish Index of Multiple Deprivation.

R^2^ = coefficient of determination, ΔR^2^ = change in coefficient of determination from step 1 to step 2.

For demographics gender (β = .15, *p* > .05), age (β = −.40, *p* > .001) and multiple deprivation (β = .18, *p* > .001) were significantly associated with physical activity in a first step. Entering intention and self-efficacy in a second step, self-efficacy (β = .27, *p* > .001, ∆*R*^*2*^ = .07) additionally explained variance in physical activity, whereas only the effect of gender decreased to non-significance. Only for the physical health domain, the social cognitive variables failed to explain additional variance. Physical functioning (β = .40, *p* > .001), bodily pain (β = −.12, *p* > .01), and FLP physical domain (β = −.21, *p* > .001) were significantly associated with physical activity in first step of the hierarchical regression and remained significant in second step, when self-efficacy (β = .002, *p* < .05) and intention (β = .07, *p* < .05) were introduced in the regression. Within the mental health domain, depression (β = −.20, *p* > .001) and anxiety (β = −.21, *p* > .001) were the dominantly related to activity in the first step, but self-efficacy explained additional variance in a second step (β = .18, *p* > .05, ∆*R*^*2*^ = .03). Looking at the social domain, need for support (β = −.12, *p* > .01), received support (β = .18, *p* > .001), but also neighbourliness (β = .10, *p* > .05) were significantly associated with physical activity in a first step, whereas only Neighbourliness remained significant, after introducing intention and self-efficacy (β = .28, *p* > .001, ∆*R*^*2*^ = .04) in a second step. In the hierarchical model for environmental variables, as well as in the model for weather variables, almost no associations to physical activity were found in the first step of regression. Only perceived personal safety was a significantly related in a first step (β = .29, *p* > .001). In both models self-efficacy (environmental model: β = .29, *p* > .001, ∆*R*^*2*^ = .05; weather model: β = .30, *p* > .001, ∆*R*^*2*^ = .13) could explain additional independent variance in physical activity.

### Social cognitions as mediators (hypothesis 2)

Overall, results indicating support for the mediation hypothesis (see Table 3). Intention and self-efficacy mediate the relationships of most domain variables with physical activity with slightly stronger effects via self-efficacy than via intention.

Looking separately within each of the domains, for demographics, there was a strong indirect mediator relationship from gender via intention (0 = women, 1 = men; β = .08, *p* > .05) and self-efficacy (β = .08, *p* > .05) in that way that men had higher levels of social cognitions and these, in turn, were associated with higher levels of physical activity. The indirect relationship from age via intention (β = .05, *p* > .05) and self-efficacy (β = .05, *p* > .05) on physical activity, but also from multiple deprivation index via self-efficacy (β = .03, *p* > .05) was significant. Only the indirect relationship from deprivation via intention was not significant (β = .03, *p* < .05). Further, strong evidence for mediation was found in the physical health domain (β = |.05| – |.12|, *p* > .05), but also for mental health (β = |.03| – |.15|, *p* > .05). For physical health the SF-36 general health - physical activity relationship was mediated through self-efficacy (β = .12, *p* > .05), for mental health, the relationship between the FLP psychosocial subscale and physical activity via self-efficacy (β = −.15, *p* > .05) were the strongest. Only the mediation of SF-38 emotional role via intention was non-significant. For the social domain, the indirect mediator relationships were substantial (β = |.05| – |.09|, *p* > .05). For both types of social support a full mediation via self-efficacy – that is the direct relationship no longer remains significant, after introducing the moderator into the model – was observed. For intention as mediator a full mediation occurred only for need of support. Within the environmental domain the effect of the indirect relationship ranged between β = .03 (*p* > .05) for traffic via intention on physical activity as the lowest but still significant indirect effect and β = .10 (*p* > .05) for personal safety via self-efficacy as the strongest indirect relationship. Finally, weather domain occurred to be unrelated to levels of intention and self-efficacy (indirect effects: β = |.01| – |.02|, *p* < .05), and also to physical activity.

### Social cognitions as moderators (hypothesis 3)

Moderation analyses (For detailed information, see Additional file 1: Table S1) for intention and self-efficacy could be confirmed only for two of the domain variables, which were multiple deprivation and physical functioning (physical health domain, SF-36 subscale). The product term for intention and deprivation (β = .08, *p* > 05; see Additional file 1: Figure S1), but also for self-efficacy and deprivation (β = .09, *p* > 05; see Additional file 1: Figure S2) was significant, indicating that there is a stronger relationship between both social cognitive variables and physical activity for participants from more affluent areas. Within the physical health domain, the product term for self-efficacy and physical functioning was significant (β = .09, *p* > 05; see Additional file 1: Figure S3), showing that within individuals with better physical functioning the association between self-efficacy and physical activity is positive and on a relatively high general activity level, whereas within individuals with poor physical functioning the association is slightly negative and on a lower level of physical activity over the full range of self-efficacy values.

## Discussion

The present study aimed to investigate whether social cognitions (i.e., intention and self-efficacy) are associated with behaviour independently from demographic, health-related, social, and environmental factors, which are external with respect to social cognitive theories, such as theory of planned behaviour. The common assumption that intention and self-efficacy fully mediate the relation of these external factors (demographic, health-related, social, and environmental) on behaviour was tested. Full mediation would support the sufficiency-hypothesis, that all variables external to the model affect behaviour indirectly through model variables only. Finally, the hypothesis that intention and self-efficacy moderate the relationship of demographic, health-related, social, and environmental factors on physical activity as an effect modifier was examined.

Regarding *Hypothesis 1*, self-efficacy explained additional variance in physical activity, controlling for demographic, mental health, social, environmental, and weather variables separately with the exception of physical health domain. This is in line with many studies, which find that self-efficacy and intention explain unique variance alongside external variables, such as age and gender [7] social [21], and environmental [13] influences. However, self-efficacy did not account for variance in physical activity when physical functioning was controlled for.

For *Hypothesis 2*, results indicating support for the mediation hypothesis, intention and self-efficacy mediate the relationship of most domain variables with physical activity. The full mediation assumption that the effect of external factors on physical activity is fully explained by social cognitive variables (Sufficiency-hypothesis [2]) was supported via self-efficacy for bodily pain, mental health, but also for need for support and received support, and for perceived quality of local area surroundings; and via intention for need for support.

These findings are congruent with McAuley et al. (e.g., [36]) who found self-efficacy as a mediator of the relationship between physical activity and health-related quality of life (measured with SF-36) for both, the physical and the mental functioning subscale. For mental health and consistent with the TPB and our findings, Manning and Bettencourt [14] reported depressive symptoms to be mediated by the TPB variables. Indeed, depression and anxiety were strongly associated with physical activity in the present sample. Although some studies did not support the mediation assumption for social support [22] we found it to be one of the strongest mediated relationships (full mediation). In our study perceived environmental factors showed weak association to physical activity. Only local area surrounding was associated with physical activity and this relationship was fully mediated by self-efficacy.

For moderation analyses for *Hypothesis 3*, intention and self-efficacy were found to moderate the relationships of two domain variables with physical activity; multiple deprivation and physical functioning. For multiple deprivation, the self-efficacy-physical activity relationship was stronger in less deprived individuals. Each increase in self-efficacy resulted in a greater increase in physical activity in affluent individuals; deprived individuals still benefited from self-efficacy, but to a smaller degree. A moderation effect in the same direction was found for intention and multiple deprivation. For physical functioning, the self-efficacy-physical activity relationship was more pronounced in individuals with higher values of physical functioning, indicating that these people, again, benefit more in terms of their physical activity level from each unit increase in self-efficacy. This finding may suggest that social cognitive approaches are less suitable for the most disadvantaged in terms of socio-economic and health resources. However, because of Type I error inflation in multiple comparisons, especially the moderation effects need replication in other studies before drawing firm conclusions.

There are a few further limitations to highlight: Despite objective measures the relationships under study were tested cross-sectionally and limit the conclusions to associations of external and TPB variables with levels of physical activity. Future studies should aim to elaborate changes in behaviour and causal relationships between variables and replicate findings with means of longitudinal and experimental designs. Furthermore we did not account for the consequences of making multiple comparisons. Adjustments for making multiple comparisons are recommended to avoid rejecting the null hypothesis too readily. These adjustments reduce the Type I error for null associations, but increase the Type II error for those associations that are not null which we tried to minimize in the present study. Since all models follow theory, correction for alpha error accumulation would be overly conservative and conclusions would reflect more the comprehensive measurement of variables in the study and the complexity of the models presented, than the observed relationships between study variables. As one cannot argue that ‘chance’ is the first-order explanation for the observed relationships in the present manuscript alpha error correction is not desirable Rothman, [37].

The present study took an interdisciplinary perspective and investigated the relationship between two social cognitions (intention and self-efficacy) and a range of demographic, health-related, social, and environmental factors on objectively measured physical activity in a large stratified cohort study. The findings of the present study have implications for intervention development. Self-efficacy has shown to be directly related to physical activity alongside demographic, mental health, social, environmental, and weather factors, which makes it a potential target for interventions. For physical health domain self-efficacy was not independently related, potentially reflecting that physical functioning in older adults may constraint *actual control* and therefore limiting the scope for self-efficacy to account for additional variability in physical activity. The finding that self-efficacy mediates the relationship of many external variables may guide the development of environmental interventions with a focus towards capacitating and encouraging older people to participate in physical activity.

Given demographic changes and the decline of physical activity levels with increasing age and higher deprivation status, determining the important factors associated with physical activity from an interdisciplinary perspective and putting social cognitive theory into context is essential to develop effective strategies to improve activity and subsequent health in old age.

## Competing interests

The authors declare that they have no competing interests.

## Authors’ contributions

FFS, METM, MDW, PTD and IKC conceived the study and thought funding for the Physical Activity Cohort Scotland (PACS) from which the data for this paper is taken. All co-authors agreed the analysis plan and PG analysed the data. PG drafted the manuscript and all co-authors provided feedback. All authors read and approved the final manuscript.

## Supplementary Material

Additional file 1: Table S1 *Estimated Moderator Analysis Coefficients on Physical Activity*. **Figure S1.** Relationship between intention and objectively measured mean daily minutes of physical activity over one week by levels of multiple deprivations. *Note*. All variables were treated as continuous and were mean centred for coefficient estimation, interaction coefficient β = .08*, *p* > 05. Intention scale is measured ranging from 1 = low to 6 = high. **Figure S2.** Relationship between self-efficacy and objectively measured mean daily minutes of physical activity over one week by levels of multiple deprivations. *Note*. All variables were treated as continuous and were mean centred for coefficient estimation, interaction coefficient β = .09*, *p* > 05. Self-efficacy scale is measured ranging from 1 = low to 6 = high. **Figure S3. **Relationship between Self-efficacy and objectively measured mean daily minutes of physical activity over one week by levels of physical functioning (SF-36 subscale). *Note*. All variables were treated as continuous and were mean centred for coefficient estimation, interaction coefficient β = .09*, *p* > 05. Self-efficacy scale is measured ranging from 1 = low to 6 = high. Click here for file
