# Supplementary material for: Psychological theory in an interdisciplinary context: psychological, demographic, health-related, social, and environmental correlates of physical activity in a representative cohort of community-dwelling older adults
Source: Int J Behav Nutr Phys Act. 2013 Sep 8;10:106. doi: 10.1186/1479-5868-10-106 (PMC3847689; doi:10.1186/1479-5868-10-106)
Supplement: Additional file 1: Table S1 — Estimated Moderator Analysis Coefficients on Physical Activity. Figure S1. Relationship between intention and objectively measured mean daily minutes of physical activity over one week by levels of multiple deprivations. Note. All variables were treated as continuous and were mean centred for coefficient estimation, interaction coefficient β = .08*, p > 05. Intention scale is measured ranging from 1 = low to 6 = high. Figure S2. Relationship between self-efficacy and objectively measured mean daily minutes of physical activity over one week by levels of multiple deprivations. Note. All variables were treated as continuous and were mean centred for coefficient estimation, interaction coefficient β = .09*, p > 05. Self-efficacy scale is measured ranging from 1 = low to 6 = high. Figure S3. Relationship between Self-efficacy and objectively measured mean daily minutes of physical activity over one week by levels of physical functioning (SF-36 subscale). Note. All variables were treated as continuous and were mean centred for coefficient estimation, interaction coefficient β = .09*, p > 05. Self-efficacy scale is measured ranging from 1 = low to 6 = high. [file 1479-5868-10-106-S1.docx]

- - - SUPPLEMANTARY TABLES AND FIGURES - - -

Supplement Table S1

*Estimated Moderator Analysis Coefficients on Physical Activity*

| Variables | Intention | | |  | Self-efficacy | | |
| --- | --- | --- | --- | --- | --- | --- | --- |
|  | *Focal predictor* | *Intention* | *Interaction* |  | *Focal predictor* | *Self-efficacy* | *Interaction* |
| Demographic |  |  |  |  |  |  |  |
| Gender (0 = women, 1 = men) | .08 | .33*** | -.04 |  | .07 | .36*** | -.03 |
| Age | -.34*** | .26*** | -.004 |  | -.33*** | .30*** | -.06 |
| Deprivation | .14** | .31*** | .08* |  | .13*** | .35*** | .09* |
| Physical health |  |  |  |  |  |  |  |
| SF-36 physical functioning | .48*** | .09* | .06 |  | .48*** | .11* | .09* |
| SF-36 role physical | .17** | .28*** | -.006 |  | .15*** | .31*** | .02 |
| SF-36 bodily pain | .08 | .29*** | -.05 |  | .06 | .33*** | -.02 |
| SF-36 general health | .21*** | .25*** | .03 |  | .18*** | .29*** | .03 |
| FLP physical domain | -.39 | .11** | .06 |  | -.37*** | .13** | .02 |
| Mental health |  |  |  |  |  |  |  |
| SF-36 vitality | .21*** | .25*** | .01 |  | .18*** | .29*** | .03 |
| SF-36 social functioning | .16*** | .29*** | .04 |  | .12* | .33*** | .02 |
| SF-36 role emotional | .04 | .32*** | .02 |  | .03 | .36*** | .03 |
| SF-36 mental health | .04 | .31*** | .03 |  | .03 | .36*** | .05 |
| FLP psychosocial domain | -.15** | .25*** | .01 |  | -.14** | .30*** | -.002 |
| Depression | -.23*** | .25*** | -.003 |  | -.21*** | .28*** | -.04 |
| Anxiety | .07* | .33*** | .04 |  | .08* | .36*** | .03 |
| Social |  |  |  |  |  |  |  |
| Need for support | -.04 | .31*** | .03 |  | -.02 | .35*** | .03 |
| Received support | .09* | .28*** | -.02 |  | .07 | .34*** | .02 |
| Loneliness | .09 | .32*** | .005 |  | .08 | .36*** | .02 |
| Neighbourliness | .10** | .31*** | .02 |  | .01** | .34*** | .04 |
| Environmental |  |  |  |  |  |  |  |
| Local area Surroundings | .08* | .31*** | .001 |  | .08* | .35*** | .03 |
| Streets in your local area | -.008 | .32*** | .019 |  | -.02 | .36*** | .02 |
| Traffic | .004 | .32*** | .005 |  | .004 | .36*** | -.04 |
| Pedestrian safety | -.03 | .32*** | .008 |  | -.04 | .36*** | -.03 |
| Personal safety | .18*** | .26*** | -.003 |  | .17*** | .30*** | .006 |
| Weather |  |  |  |  |  |  |  |
| Sun (hours) | .07 | 32.*** | .02 |  | .07 | .35*** | .002 |
| Minimum temperature | .01 | .32*** | .01 |  | .02 | .35*** | .01 |
| Maximum temperature | .04 | .32** | .02 |  | .04 | .35*** | .02 |
| Rainfall | -.04 | 31.*** | .01 |  | -.05 | .35 | -.01 |

*Note*. *N* = 574. Focal predictor refers to the coefficient of the variable in the first column of the Table on physical activity, controlled for intention *or* self-efficacy and the product term of intention *or* self-efficacy and the focal predictor.


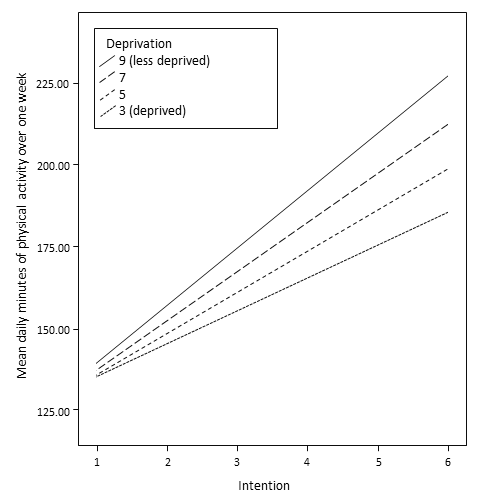


*Supplement Figure 1.* Relationship between intention and objectively measured mean daily minutes of physical activity over one week by levels of multiple deprivations.

*Note.* All variables were treated as continuous and were mean centred for coefficient estimation, interaction coefficient β = .08*, *p* > 05.


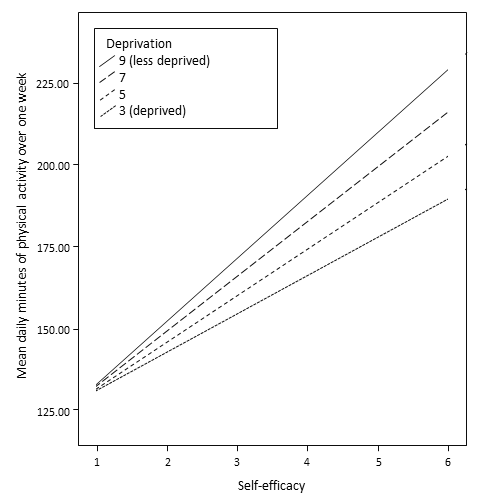


*Supplement Figure 2.* Relationship between self-efficacy and objectively measured mean daily minutes of physical activity over one week by levels of multiple deprivations.

*Note.* All variables were treated as continuous and were mean centred for coefficient estimation, interaction coefficient β = .09*, *p* > 05.


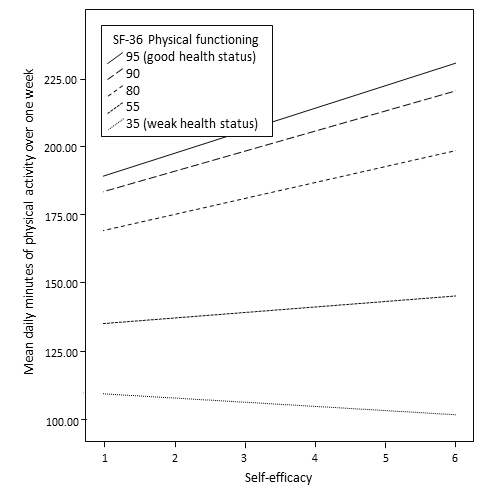


*Supplement Figure 3.* Relationship between Self-efficacy and objectively measured mean daily minutes of physical activity over one week by levels of physical functioning (SF-36 subscale).

*Note.* All variables were treated as continuous and were mean centred for coefficient estimation, interaction coefficient β = .09*, *p* > 05.
